# Usage, Unmet Needs, Barriers, and Satisfaction With Accessing Assistive Technology Among Persons With Disabilities: Findings From a Tertiary Care Hospital in North India

**DOI:** 10.7759/cureus.105418

**Published:** 2026-03-18

**Authors:** Ganesh Yadav, Suman Badhal, Ravindra Kumar, Nonica Laisram, Isha Preet Tuli, Lopamudra Sarkar

**Affiliations:** 1 Physical Medicine and Rehabilitation, Vardhman Mahavir Medical College and Safdarjung Hospital, New Delhi, IND; 2 Otorhinolaryngology, Vardhman Mahavir Medical College and Safdarjung Hospital, New Delhi, IND; 3 Ophthalmology, Vardhman Mahavir Medical College and Safdarjung Hospital, New Delhi, IND

**Keywords:** assistive products, assistive technology, barriers, needs, persons with disability, satisfaction, unmet needs, usage, who rata tool

## Abstract

Objective: This study aimed to estimate usage, unmet needs, barriers, and satisfaction related to access to assistive technology (AT) among outpatients at a tertiary care hospital in North India.

Methods: This single-center, prospective observational cross-sectional study was conducted in the outpatient departments of physical medicine and rehabilitation, ophthalmology, and otorhinolaryngology at Safdarjung Hospital, New Delhi, India. Data were collected using the World Health Organization’s (WHO) rapid Assistive Technology Assessment (rATA) tool. Participants were recruited consecutively from the respective outpatient departments, using population-proportion sampling to ensure adequate representation.

Results: A total of 550 patients were surveyed. The need for AT was identified in 536 participants, with a mean age of 38.83 ± 14.69 years. Unmet AT needs were significantly higher among participants from lower socioeconomic status compared to those from upper socioeconomic status (chi-square (χ²) = 12.13, p < 0.001), among youth and middle-aged adults (18-59 years) compared to elderly participants (>60 years) (χ² = 21.56, p < 0.001), and among the working population compared to non-working participants (χ² = 9.88, p < 0.001). No statistically significant association was observed between AT need and unmet need with gender, educational status, area of residence, or severity of difficulty/functional limitation.

Conclusion: This study demonstrates substantial unmet needs for AT, driven by socioeconomic disparities, affordability barriers, and limited access. Strengthening awareness, expanding equitable national coverage of assistive products, and implementing standardized assessment tools such as the WHO's rapid rATA are essential to bridging the gap between demand and availability. These measures are critical to improving access to AT for individuals with disabilities and functional impairments.

## Introduction

Assistive technology (AT) has been receiving growing international interest over the past few years as a vital part of healthcare and well-being. It is important in enhancing the functional capacity and independent living of persons with functional limitations or disabilities. To enhance access to AT for people throughout the world, the World Health Organization (WHO) created the Global Cooperation on Assistive Technology (GATE) initiative, which established the Priority Assistive Products List (APL) of 50 key assistive products to enhance the lives of individuals with various functional challenges. Disability is one of the biggest global health issues, which influences the engagement of people in routine life, education, and work. In India, disability burden represents the necessity to provide rehabilitation services and ATs that are readily available. Shedding more weight on policy support are the Rights of Persons with Disabilities Act, 2016, and programs like the Assistance to Disabled Persons to Purchase/Fit Aids and Appliances Scheme (ADIP), which have enhanced the support of the policy. Nevertheless, there are still areas of insufficient awareness, access, and efficient use, which highlight the importance of better AT delivery mechanisms and evidence-based approaches [1-3]. Instead of requiring complex, esoteric engineering, AT is a way of thinking that seeks solutions to the problems that persons with disabilities face daily. There are three levels of AT: Low-tech alternatives are simple to use, inexpensive, and normally do not need a power source; med-tech options are simple to operate but typically do need a power source, and high-tech options are frequently sophisticated and programmable and include devices that need computers and/or electronics to function. Others add a further level known as "no-tech," which is the adaptation of behavior or technique of communication, such as speaking less and employing gestures [3]. ATs are vital for persons with disabilities because they facilitate independent living and participation in social and daily activities. Evidence indicates that use of AT helps to minimize functional limitations associated with mobility, cognition, self-care, hearing, and vision, and assists the individual to overcome the challenges in both academic and non-academic occupational performance [2-5]. Based on the reduction of disability in healthy living, productive independence, and dignity, assistive products such as wheelchairs, white canes, hearing aids, communication aids, vision aids, and other ATs promote the quality of life. They promote participation in education, employment, and community activities; provide preventative support for additional impairments; and reduce the burden on formal health care, long-term care utilization, and family caregiver time [6].

The WHO Global Report on Assistive Technology (GReAT) reports vast global inequalities in access to AT, from around 3% unmet in low- and middle-income countries (LMICs) to almost 90% coverage in high-income countries. Despite all the promises, policies, and actions of the government, only 10% of the needs of the people with hearing impairment in the world are being met [6,7].

Recent estimates from the WHO indicate that approximately one in three people worldwide requires at least one assistive product to address functional limitations. While the need for AT increases with age, individuals with disabilities remain the most vulnerable group and have the greatest unmet need for such support. Considering current demographic trends, the global demand for AT is expected to increase to “3.5 billion by 2050” [7].

Limited access to AT can negatively impact health by increasing the risk of secondary conditions and restricting access to healthcare, with broader effects on families and communities. Over time, this contributes to a greater burden on healthcare systems, particularly in LMICs. Failure to adequately include persons with disabilities in social and economic systems can result in economic losses of up to 7% of a country’s gross domestic product. This estimate has been reported by the World Economic Forum and related economic analyses on disability inclusion [8]. Thus, investment in AT and rehabilitation services has significant societal and economic benefits. Limited access to AT restricts the participation of persons with disabilities in education, employment, and community life, which can reduce workforce productivity and increase dependence on healthcare and social support systems. Since the 71^st^ World Health Assembly in 2018, the WHO, through initiatives such as GATE, has advocated for improved access to quality and affordable AT across its member states [9].

Simultaneously, United Nations Sustainable Development Goals (SDGs) goal 3.8, access to AT, is a crucial part of the continuum of health care and emphasizes the importance of social inclusion, pledging that “no one should be left behind.” Governments are encouraged to prioritize efforts to identify and support populations that remain underserved. As a ratified member of both the WHO and the United Nations, India is dedicated to being on par with other global efforts to develop AT initiatives that improve access to safe, quality, and affordable AT [10]. In India and other countries, especially in South Asia, although the exact magnitude of the needs for ATs and unmet needs is not fully known, demand is already high and expected to rise further due to ongoing demographic and epidemiological transitions. According to the 2011 national census, approximately 2.2% of the population, about 30 million people, were living with some form of disability, highlighting the scale of potential need [11]. However, as the living standard and health facilities are increasing and improving day by day, there is an increase in the effective years of life. Consequently, an increasing number of individuals with functional limitations or disabilities require AT, and this enables their integral and community participation in various activities on par with normal individuals. According to WHO estimates, around a billion individuals (or 15% of the world's population) are disabled, and between 110 million (2.2%) and 190 million (3.8%) adults aged 15 and older struggle with serious functioning issues [12].

Estimates of the prevalence of disability in India based on the 2011 Census have come out to be substantially lower than those of international agencies like the WHO, the World Bank, etc. World Bank estimates from 2007 suggest that disability prevalence in India may be at least 1.5 times higher than census estimates and potentially up to three times higher when a broader range of disabilities is considered. This discrepancy underscores the need for more robust research and reliable data to accurately determine the true magnitude of need and unmet needs for AT in the country. Such variation may partly reflect differences in methodological approaches, definitions of disability and AT needs, data sources, and assessment methods used across studies [13,14].

The information regarding unmet needs, demand, impediments, and patterns of access to assistive products, despite the growing interest in assistive products in the health, disability, and social spheres, is not readily available [15]. There is a need to have reliable data regarding who is at risk of not accessing the services they need and the barriers to access, so as to develop effective plans to scale up assistive product services. To compensate for this deficiency of high-quality data to inform practice and policy in assistive product services, the WHO has created a rapid Assistive Technology Assessment (rATA) questionnaire that can be performed in quick and comprehensive assessments of the need, unmet need, demand, supply, and satisfaction impact of AT in the community [16]. The rATA questionnaire contains the following issues: personal report on the use and demand of assistive products; the sources of, prayers about, and obstacles to assistive products and related services; personal report on their satisfaction and dissatisfaction with assistive products and related services; personal issues in terms of their functional problems; and personal factors including age, gender, etc. [16].

The present study aims to assess the self-reported need and unmet need for AT, barriers to access, and user satisfaction with assistive products among individuals attending a tertiary care hospital in North India.

## Materials and methods

Survey tool

The WHO rATA tool (version 4, 3rd July 2021) indicators were facility-based and comprised of five components: (A) preliminary information/administrative data, (B) demographics, (C) need, (D) demand and supply, sources of assistive products, payers of assistive products, distance to assistive products facility, unmet needs, and barriers to access, and (E) satisfaction. The tool is pre-validated and was forward- and backward-translated by language experts. The selected study participants were interviewed for sociodemographic details and the WHO rATA tool [16] questionnaire. Modified Kuppuswamy's socioeconomic scale (2020) was used for the socioeconomic status of study participants [17].

Ethical approval

This study was initiated after obtaining approval from the Institutional Ethics Committee of Vardhman Mahavir Medical College and Safdarjung Hospital, New Delhi, India (IEC/VMMC/SJH/Thesis/2020-11/CC-267) dated 10/12/2020.

Sample design and population

This single-center, prospective observational cross-sectional study included persons with disabilities and functional impairment and aimed to assess the self-reported need and unmet need for AT, barriers to access, and user satisfaction with assistive products among individuals attending a tertiary care hospital in North India with visual, hearing, and locomotor disabilities. Participants were recruited from the outpatient departments of physical medicine and rehabilitation, ophthalmology, and otorhinolaryngology at a tertiary care hospital in New Delhi between February 2021 and June 30, 2022. The inclusion criteria were as follows: patients who were 18 years or above, having locomotor, visual, or hearing disability. The study excluded patients who had an inability to speak or understand, and those with profound hearing loss who were not using a hearing aid.

To understand end-user experiences, persons with disabilities and functional impairments aged 18 years and above who met the inclusion criteria were enrolled in the study.

Data collection and sampling method

Data were collected from the outpatient departments of three distinct specialties: physical medicine and rehabilitation, ophthalmology, and otorhinolaryngology. The records were obtained from the Medical Record Section. According to old records from past years, the highest percentage was from physical medicine and rehabilitation, followed by ophthalmology and otorhinolaryngology. Our sample was divided into strata of PMR, ophthalmology, and otorhinolaryngology. Using population proportion sampling based on old records, we determined the desired sample size for each department. 285, 154, and 111 study participants from physical medicine and rehabilitation, ophthalmology, and otorhinolaryngology were recruited, respectively. The sampling technique was consecutive sampling.

Sampling calculation

In order to come up to a sample size, we took into consideration the unmet need of AT among people with disabilities to be 71%, as reported by Pryor et al. [18], an error value of 6% and a confidence level of 95%. It is in these parameters that the estimated sample size was described as 436.

The Cochrane formula was used as follows:

$(Z_{\alpha})^2 \times P \times (100 - P) / L^2$

P =71% and L (relative error) =4.2, and since we want to add an error of 25% to it, we have 545 (the round off) =550 patients.

Data management and analysis

The data were entered into Microsoft Excel (Microsoft Corp., Redmond, WA, USA) and checked for errors before analysis was done. Descriptive statistics, including means and percentages, were used to summarize the results. Participant identification numbers were applied instead of names for confidentiality. For categorical variables, frequency and proportion were used in the visual analysis. For continuous variables, descriptive statistics in terms of minimum, maximum, and mean (± standard deviation) were presented. Cross-tabulation between categorical variables was tested through the chi-square (χ²) test, and if the p-value is 0.05 or less, it was considered statistically significant. For the statistical analysis, IBM SPSS software, version 21 (IBM Corp., Armonk, NY, USA), was used.

Study definitions

Assistive Products

Assistive products were defined as externally used products that support one's functional ability and independence without therapeutic or curative intent. Product identification was done from the standardized list included in the WHO rATA tool, along with visual "AT Show Cards" to facilitate accurate reporting by participants [19].

Assistive Technology

Assistive products, along with associated service provision systems such as assessment, prescription, fitting, user training, and follow-up, are provided to individuals who require AT. These services enable users to use assistive products safely, effectively, and for their intended purpose. In line with the WHO framework, assistive products are intended for individuals experiencing functional difficulties or limitations in performing everyday activities [19].

Use of AT

It refers to the percentage of individuals currently using one or more assistive products among the total number of participants included in the study [19].

Need for AT

The need for AT refers to the proportion of individuals who require one or more assistive devices to address their functional limitations. This measure includes both met needs and unmet needs [19].

Met Need for AT

Met need refers to the proportion of individuals who require assistive products and currently possess the appropriate devices, without the need for additional or replacement products or services for the specific functional difficulty at the time of the study [19].

Unmet Need of AT

Unmet need refers to the proportion of individuals who require new or additional assistive products to address a specific functional difficulty. This includes individuals who lack access to a required device or who have a device that is broken, inappropriate, or otherwise inadequate and requires replacement [19].

Barriers to Accessing AT

Barriers refer to the difficulties encountered by individuals who do not have access to the assistive products they need, as identified in the study [19].

## Results

Demographic features of the study participants

The study was conducted on 550 participants who were evaluated using the WHO rATA tool. The mean age of the respondents was 38.83 years with SD = 14.69. Men accounted for almost two-thirds of the sample (65.1, n=358), and 46.2% (n=254) were living in urban settings (Table 1). Individuals between 25 and 59 years of age formed the largest age group (65.6%, n = 361), followed by those aged 18-24 years (23.1%, n = 127). Most participants had attained formal education at some level (86.5%, n = 476), and just over half were engaged in employment (52.9%, n = 291). Functional difficulties related to mobility were reported most frequently (68.2%, n = 375). Challenges with self-care were also common (57.3%, n = 315), while visual impairments were reported by 35.8% of participants (n = 197) (Table 1).

**Table 1:** Demographic Characteristics of the Study Participants (N = 550) *Modified Kuppuswamy Scale (2020) [17]

Variables	Summary statistics
Mean (SD) age, years	38.83 (14.69)
Age group	
Youth (18 to 24 years)	127 (23.1%)
Older adults (25 to 59 years)	361 (65.6%)
Elderly (≥60 years)	62 (11.3%)
Gender	
Male	358 (65.1%)
Female	192 (34.9%)
School attended	
Never attended	74 (13.5%)
Ever attended	476 (86.5%)
Working status	
Not working	259 (47.1%)
Working	291 (52.9%)
Socioeconomic status*	
Lower	16 (2.9%)
Upper lower	268 (48.7%)
Lower middle	97 (17.6%)
Upper middle	166 (30.2%)
Upper	3 (0.5%)
Area of residence	
Rural	101 (18.4%)
Semi-urban	195 (35.5%)
Urban	254 (46.2%)
Functional difficulties	
Mobility	375 (68.2%)
Seeing	197 (35.8%)
Hearing	119 (21.6%)
Communicating	22 (4%)
Remembering	11 (2%)
Self-care	315 (57.3%)
Any difficulties	546 (99.3%)

Nearly all participants (99.3% (n = 546); Table 1) reported at least one difficulty across the assessed domains, with the majority experiencing severe or complete difficulties (92.4% (n = 508); Table 2). Among participants reporting any difficulty, the highest prevalence was observed in the self-care domain (38.7% (n = 213)), followed by mobility (20.9% (n = 115)) and visual impairments (6.7% (n = 37)). Overall, difficulty severity was categorized as none, some, or severe/total, with 0.7% (n = 4) reporting no difficulty, 6.9% (n = 38) reporting some difficulty, and 92.4% (n = 508) reporting severe or total difficulty. Severe or total difficulties were most commonly reported in the mobility domain (47.3% (n = 260)), followed by visual (29.1% (n = 160)) and hearing impairments (20.7% (n = 114)) (Table 2).

**Table 2:** Severity of Functional Difficulties Across Domains (N = 550)

Difficulties	No difficulty n (%)	Some difficulty n (%)	Severe/total difficulty n (%)
Any difficulties	4 (0.7%)	38 (6.9%)	508 (92.4%)
Mobility	175 (31.8%)	115 (20.9%)	260 (47.3%)
Seeing	353 (64.2%)	37 (6.7%)	160 (29.1%)
Hearing	431 (78.4%)	5 (0.9%)	114 (20.7%)
Communicating	528 (96%)	18 (3.3%)	4 (0.7%)
Remembering	539 (98%)	11 (2%)	0 (0%)
Self-care	235 (42.7%)	213 (38.7%)	102 (18.5%)

Met and unmet needs for AT

**Figure 1:**
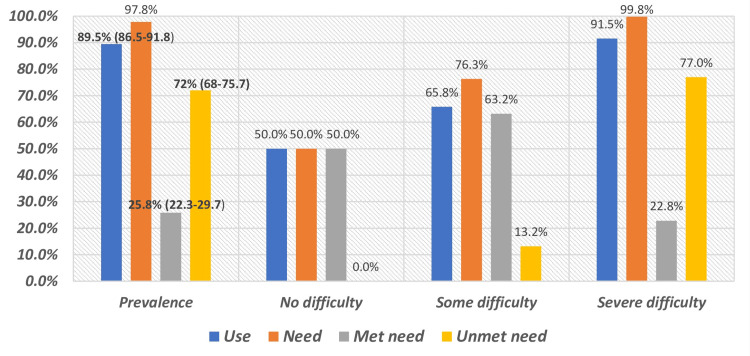
Use, Need, and Unmet Need for Assistive Technology and Associated Functional Difficulties

The rATA questionnaire used by WHO evaluates unmet need using the question: "Do you think you need an assistive product(s) that you do not currently use, or that you currently use but need to be replaced?" The estimated proportion of participants requiring AT was 72% (Figure 1), which closely corresponded with the observed sample proportion of 73.8% (n = 396; Table 3). The proportion of met AT needs varied by the severity of functional difficulty, ranging from 26.1% (n = 140) among participants with severe difficulties to 50% (n = 2) among those reporting no difficulties (Table 3).

The overall estimated unmet need for AT was 72% (n = 386). Unmet need was observed in 73.8% (n = 396) of participants with severe or total functional difficulties (Figure 1; Table 3). Participants residing in rural areas reported a higher prevalence of unmet needs than those from semi-urban areas (80.4% (n = 78) vs. 69.2% (n = 133); Table 3).

**Table 3:** Assistive Technology Need, Usage, Met Need and Unmet Need in Relation to Participant Characteristics © n defines need, and that can be multiple entries per individual; Chi-square (χ²) tests were used to compare categorical variables across age groups, gender, school attended, working status, area of residence, and socioeconomic status. Fisher’s exact test was used for the difficulties domain; *Statistically significant at P-value <0.05; *Modified Kuppuswamy scale (2020) [17]

Variables (n=536) ©	Met need	Unmet need	Test Statistics	Usage
Age group				
Youth (18 to 24 years) (n=123)	26 (21.1%)	97 (78.8%)	χ² = 21.562, P-value <0.001*	109 (88.6%)
Older adults (25 to 59 years) (n=354)	85 (24.0%)	269 (75.9%)		328 (92.6%)
Elderly (≥60 years) (n=61)	31 (50.8%)	30 (49.1%)		55 (90.1%)
Gender				
Male (n=348)	91 (26.1%)	257 (73.8%)	χ² = 0.0304, P-value 0.861692	319 (91.6%)
Female (n=190)	51 (26.8%)	139 (73.1%)		173 (91.0%)
School attended				
Never attended (n=73)	18 (24.6%)	55 (75.3%)	χ² = 0.1311, P-value 0.717298	61 (83.5%)
Ever attended (n=465)	124 (26.6%)	341 (73.9%)		431 (92.6%)
Working status				
Not working (n=254)	51 (20.0%)	203 (79.9%)	χ² = 9878, P-value <0.001*	228 (89.7%)
Working (n=284)	91 (32.0%)	193 (67.9%)		264 (92.9%)
Socioeconomic status*				
Lower (n=275)	56 (20.3%)	219 (79.6%)	χ² = 12.1345, P-value <0.001*	234 (85.0%)
Upper (n=263)	86 (32.6%)	177 (67.3%)		258 (98.0%)
Area of residence				
Rural (n=97)	19 (19.5%)	78 (80.4%)	χ² =4.2316, P-value 0.12053	87 (89.6%)
Semi-urban (n=192)	59 (30.7%)	133 (69.2%)		173 (90.1%)
Urban (n=249)	64 (25.7%)	185 (74.2%)		232 (93.1%)
Functional difficulties				
Mobility (n=367)	62 (16.8%)	305 (83.1%)		348 (94.8%)
Seeing (n=195)	64 (32.8%)	131 (67.1%)		182 (93.3%)
Hearing (n=119)	32 (26.8%)	87 (73.1%)		92 (77.3%)
Communicating (n=22)	12 (54.5%)	10 (45.4%)		20 (90.9%)
Remembering (n=11)	7 (63.6%)	4 (36.3%)		11 (100%)
Self-care (n=313)	47 (15.0%)	266 (84.9%)		303 (96.8%)
Any difficulties (n=536)	140 (26.1%)	396 (73.8%)		490 (91.4%)
Difficulties				
No (n=4)	2 (50%)	0 (0%)	Fisher’s exact test = 0.069, P-value > 0.05	2 (50%)
Severe (n=536)	140 (26.1%)	396 (73.8%)		490 (91.4%)

Unmet needs were significantly higher among non-working participants compared to working participants (79.9% (n = 203) vs. 67.9% (n = 193), p <0.001) and among those from lower socioeconomic groups compared to the upper socioeconomic class (79.6% (n = 219) vs. 67.3% (n = 177), p <0.001). Although overall AT needs were similar across functional domains, unmet needs were most pronounced in self-care (84.9% (n = 266)) and mobility (83.1% (n = 305)) domains (Table 3).

**Figure 2:**
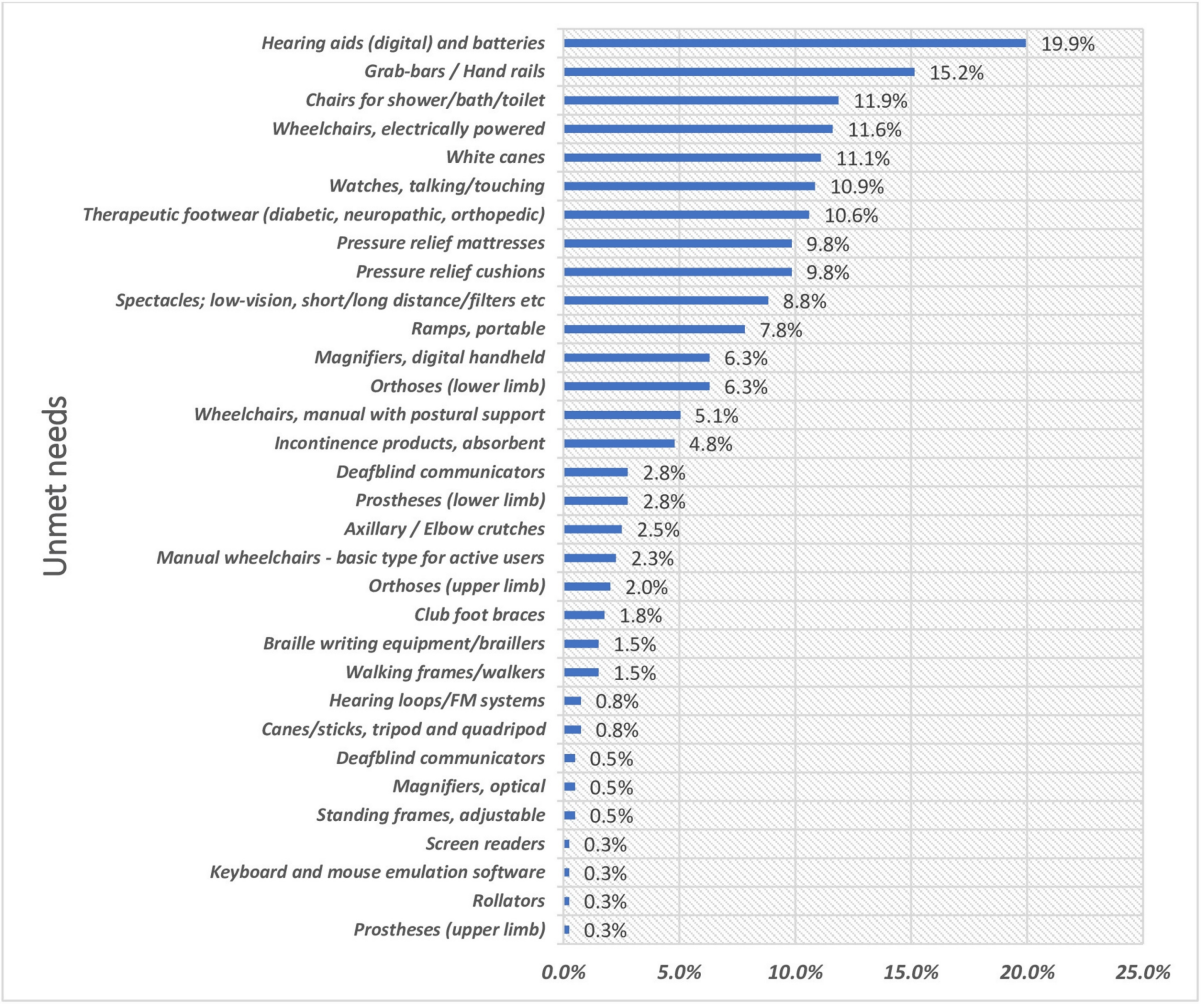
Interview-Based Self-Reported Unmet Needs for Assistive Products (N = 396)* *Multiple entries

Interview-based self-reported unmet needs were comparable across gender, with similar prevalence among males (73.8% (n = 257)) and females (73.1% (n = 139)). Unmet need for AT decreased significantly with increasing age, while met need increased correspondingly (p < 0.001; Table 3). The highest unmet needs were reported for digital hearing aids (19.9% (n = 79)), followed by grab bars/handrails (15.2% (n = 60)) and shower/bath/toilet chairs (11.9% (n = 47)) (Figure 2).

Usage characteristics of AT

Among the study participants, 89.5% reported using one or more assistive products, with comparable usage across genders (Figure 1; Table 3). As summarized in Table 3, assistive product use was consistent across age groups, from young to older adults. The highest usage was observed in the self-care domain, reported by 96.8% of participants (n = 303). Assistive product utilization differed significantly by socioeconomic status, with higher usage in the upper socioeconomic group (98% (n = 258)) compared to the lower group (85% (n = 234)) (Table 3).

**Figure 3:**
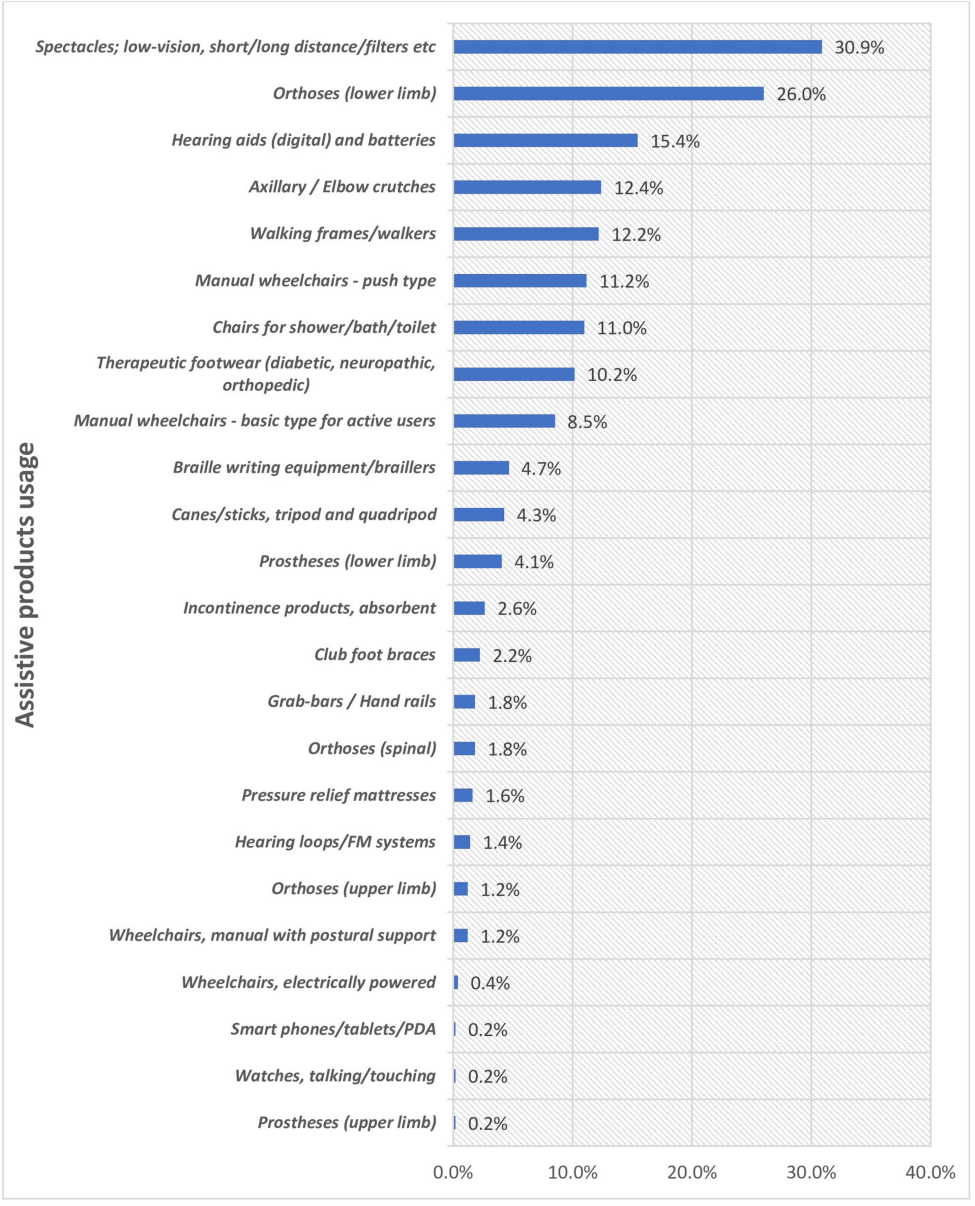
Usage of Assistive Products Among Study Participants (N = 492) *Multiple entries FM: frequency modulation; PDA: personal digital assistant

Spectacles were the most commonly used AP, accounting for 30.9% of overall use, followed by lower limb orthoses (26% (n = 128)) and hearing aids (15.4% (n = 76)) (Figure 3).

User satisfaction and barriers to accessing AT

**Figure 4:**
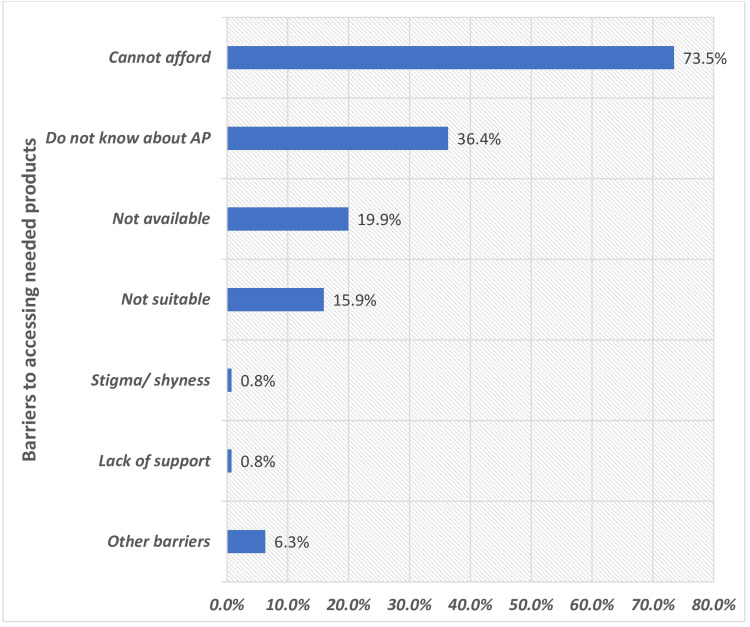
Out of the 492 Study Participants Using One or More Assistive Products (APs), 396 Reported Barriers to the Utility of Their APs, Indicating a Interview Based Self-Reported Unmet Need* Other barriers include those not captured by the World Health Organization’s (WHO) Rapid Assistive Technology Assessment (rATA) tool. *Multiple entries

The most commonly reported barrier to assistive product use was the inability to afford them, cited by 73.5% of participants (n = 291) (Figure 4). This was followed by a lack of awareness regarding assistive products (36.4% (n = 144)) and the unavailability of assistive products (19.9% (n = 79)). Additional barriers included discomfort or unsuitability of assistive products for participatory or public activities (15.9% (n = 63)) and stigma or shyness associated with AT use (0.8% (n = 3)) (Figure 4).

**Figure 5:**
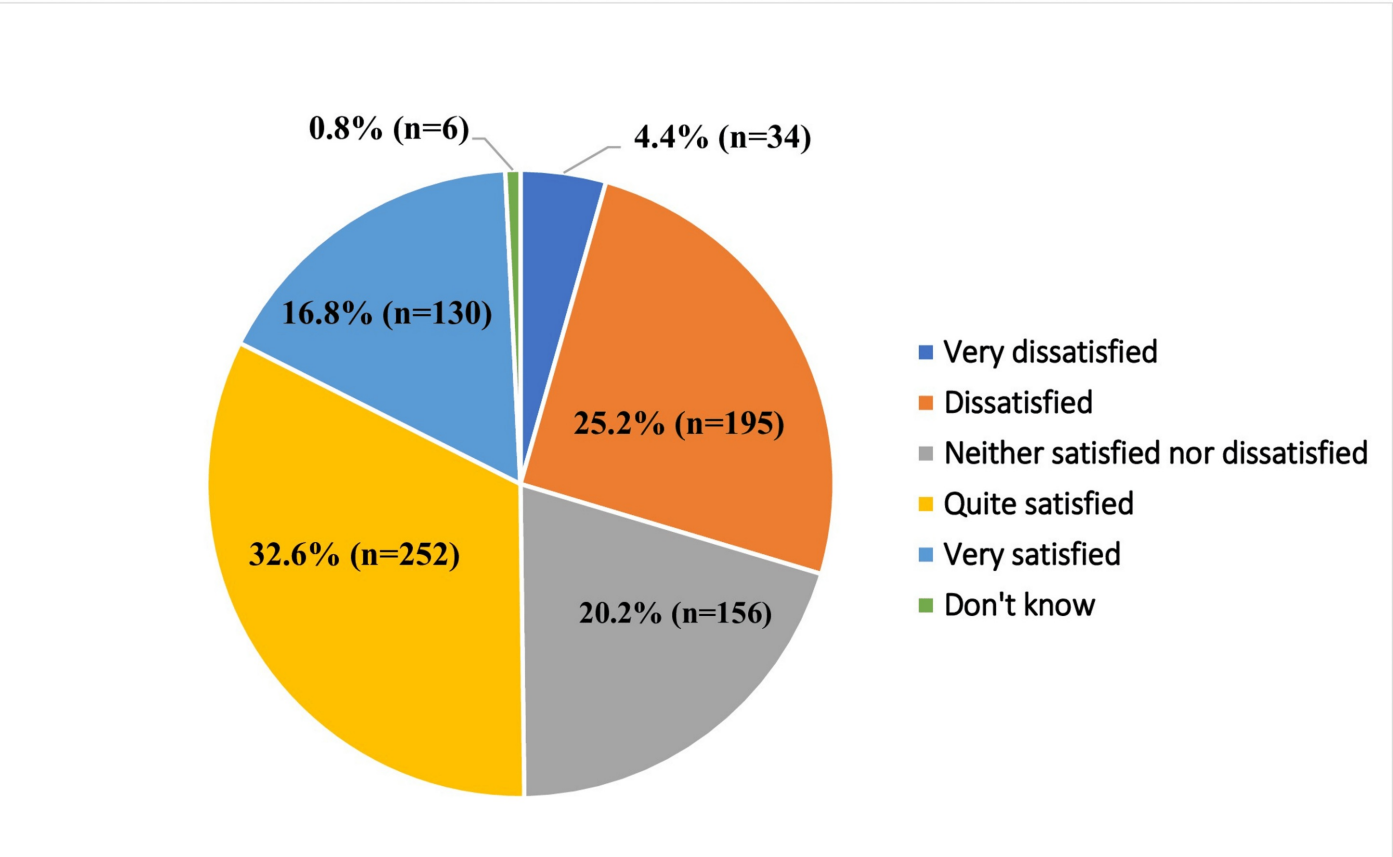
Satisfaction with Assistive Products Among Study Participants (N = 773)* *Multiple entries

Regarding satisfaction with assistive products, 49.4% of participants (382/773) reported being “quite” or “very” satisfied, while 25.2% (n = 195) reported dissatisfaction. A further 20.2% (n = 156) reported neutral satisfaction, indicating neither satisfaction nor dissatisfaction (Figure 5).

**Figure 6:**
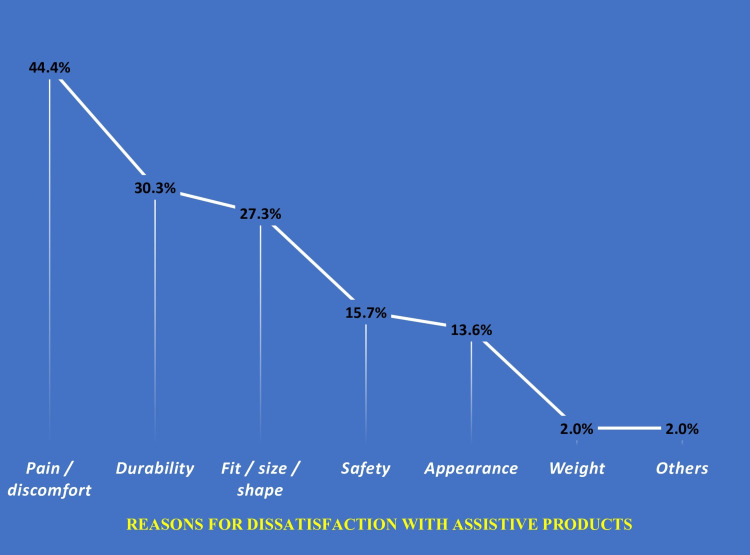
Interview-Based Self-Reported Reasons for Dissatisfaction With Assistive Products Among Study Participants (N = 198)* *Multiple entries

Among dissatisfied users, the most commonly reported reason was pain or discomfort associated with the assistive product (44.4% (n = 87)), followed by concerns related to durability (30.3% (n = 60)), improper fit, size, or shape (27.3% (n = 54)), and safety issues (15.7% (n = 21)). Excessive weight was infrequently reported (2% (n = 4)) (Figure 6).

## Discussion

The ongoing demographic transition is driving a substantial increase in the elderly population, as well as in individuals living with chronic health conditions and various disabilities worldwide. The WHO estimates there are currently more than one billion people with disabilities worldwide, and that number is projected to grow to nearly two billion by the year 2030. This expanding population highlights the growing demand for AT to support functioning, promote independence, and enhance overall well-being across diverse populations [14].

To the best of our knowledge and based on our literature review, we did not come across any hospital-based studies; most of the research has been conducted at the community level. In our tertiary care hospital study, despite good access to one or more assistive products among participants, high unmet needs were associated with a better understanding of the potential benefits of assistive products, combined with the poor performance of low-cost products and inadequate maintenance and repair services for assistive products. These findings are consistent with those of the study by Pryor et al. [18]. The findings suggest that key study indicators, including the proportion of met need and actual use of AT, increase with advancing age. However, despite this trend, the level of unmet need for AT observed in the present study remains higher than that reported in neighboring countries, where previous estimates have documented unmet needs of 13.1% in Pakistan and 51% in Bangladesh. This disparity may reflect differences in healthcare infrastructure, availability of assistive products, awareness, and socioeconomic factors, underscoring the urgent need to strengthen AT provision and accessibility in the local context (as shown in Table 4 and Figure 1) [17]. This result may be attributed to the fact that our study population consisted exclusively of individuals seeking medical attention for functional impairments at a tertiary care hospital, rather than a representative sample from the general community. Additionally, India, home to 18% of the world’s population, is experiencing a rapid increase in its elderly population, many of whom are affected by non-communicable diseases, further contributing to the demand for AT [20].

**Table 4:** Results of Rapid Assistive Technology Assessment (rATA) Studies in Other Countries and Tertiary Care Hospital in North India * Medium-income group (MIG) countries only.

Country/region	Year	Sample size	Unmet need	Met need	Usage	Satisfaction with assistive products used
India [21]	2023	8422	8.0% (7.4-8.6)	16.5% (15.7-17.3)	19.9% (19.0-20.7)	-
Sierra Leone [22]	2019	2076	­ -	-	14.9%	-
Indonesia [22]	2019	2046	17.5%	-	47.4%	-
Bangladesh [23]	2021	11,187	51%	1.0%	11.0%	53%
Pakistan [24]	2021	62,723	13.1%	-	7.2%	-
Brazil [25]	2022	926	82.4%	48.5%	72.9%	75%
The WHO Global Report on Assistive Technology (GReAT) report* [6]	2022	9 MIG countries	-	-	33.2% (15.7-65.3)	-
Current study (North Indian tertiary care hospital)	2022	550	72% (68-75.7)	25.8% (22.3-29.7)	89.5% (86.5-91.8)	49.4% (45.8-53)

Of the domains of functional difficulty measured, mobility, vision, and hearing impairment were the most common interview-based self-reported domestic functional impairments and disabilities. Mobility difficulty was found in 375 (68.2%), vision impairment was discovered in 197 (35.8%), and hearing impairment in 119 (21.6%) people, as illustrated in Table 1. These proportions are higher than those reported in the WHO GReAT report [6], where the prevalence of mobility, vision, and hearing impairments are 12%, 20.9%, and 4.9%, respectively. Similarly, a study conducted by Senjam et al., which was community-based and was performed in eight districts of India using the WHO rATA tool, reported lower prevalence rates of impairment in terms of mobility (11.9%), vision (26.6%), and hearing impairments (3.4%) [21]. In contrast, severe and complete functional difficulties ranging from 4.3 to 7% were reported in rATA studies from Sierra Leone and Indonesia [22], which were much less than in the present study (77%), as shown in Figure 1 and Table 4. This difference is likely related to the nature of the study. People with pre-existing functional limitations are assessed, and mostly severe and complete difficulties are assessed.

Individuals with functional impairments, as well as other vulnerable populations, need AT and rehabilitation services to cope with their functional limitations and help them achieve their independence, health, and well-being. A recent joint report by the WHO and United Nations Children’s Fund (UNICEF) estimated that over 2.5 billion people worldwide currently require one or more assistive products, with this number likely to rise to 3.5 billion by 2050. Of special note, it is estimated that nearly 90% of the population in need will live in LMICs [7]. These findings highlight the need for healthcare systems to address gaps in the availability, access, and delivery of essential assistive products and associated services.

The WHO rATA tool has been implemented previously in several countries, including Pakistan, Bangladesh, Indonesia, Cameroon, Sierra Leone, and Brazil [22-25]. Although the tool is mainly developed for self-reporting, a read-aloud strategy was used in the current study in order to accommodate participants who were illiterate or had visual or hearing impairments. Questionnaires were administered using the local language, and visual "show cards" (from the Assistive Product List (APL) representing the 50 assistive products) were used for each participant to minimize reporting inconsistencies [26]. This methodology allowed for the inclusion of persons with sensory impairments, as well as the collection of reliable data.

The present study was conducted in a tertiary care hospital in North India to assess the AT needs of adults aged 18 years and above and the unmet needs, barriers, and satisfaction with access to AT using the WHO rATA tool. The tool, which was developed under the aegis of the WHO GATE initiative, has been validated for use in population-based surveys by WHO member states [26]. Minor modifications were made in our study into the demographic section to improve applicability within a tertiary-level healthcare setting.

Estimates of the need not met with AT made in the current study were 72%. This result indicates that not everyone who had problems declared the need to use AT because it is low compared to the total number of difficulties (Figure 1). Nevertheless, it should be observed that the AT need found in the given research was almost within the range of the AT need found by WHO GReAT (9.9%-68.9%), which implies that the prevalence of the AT need is not out of range compared to another study that produced similar results, such as the ones in Brazil and Bangladesh [6,23,25 ]. There were similarities in the most frequently utilized assistive products by persons with disabilities and persons with functional impairments in our research. Most of them wore spectacles; similar results were discovered in the rATA study carried out in Bangladesh on the Rohingya community living in refugee camps, GReAT statistics, and Senjam et al. (Table 4) [6, 21, 23].

The study also recognized several significant predictors of AT usage, encompassing male gender, older adulthood, literacy, employment status, higher socioeconomic status, urban residency, and experiencing any form of functional difficulty. These factors were observed to impact the likelihood of individuals adopting AT to address their functional challenges, reinforcing previous research concerning the associations between assistive product availability, affordability, and utilization [27].

It was also found that elderly people in rural areas were found to have a very high unmet AT need. It was found that the sample proportion of unmet need of AT was 72% with a slightly higher proportion in males (73.8%), youth (78.8%), rural populations (80.4%), not working/financially dependent persons (79.9%), and persons of lower socioeconomic groups (79.6%) (Table 3). It is estimated that the needs of persons with severe difficulties are not fulfilled in 77% of cases. This means that every fourth such person will not be able to access the AT that he or she needs. Results mostly correlate with the estimates of unmet needs of AT and hindrances to accessing them in assistive products of similar research (Table 4) [3, 26-29].

In spite of the fact that the WHO rATA tool was helpful during this study, it possesses significant limitations. This interview-based self-reporting would most probably lead to over- or underreporting of assistive product needs and unmet needs. Lack of knowledge and awareness of assistive products may be a reason for under-reporting, and the use of assistive products is less important. Under-detection is caused by self-reporting, participation hindrance, clinical impairments [25], excessive needs, and unmet needs, which are probable to occur where deformities or defects can be cured. Thus, further research is required to understand the accuracy and usefulness of the WHO rATA tool and other ways of gaining a better insight into the needs, unmet needs, and the demand for assistive products by people with disabilities. Although our results largely agree with others, the patterns of access and provision are most likely to be highly situational and may not be generalized into other contexts.

The research also concluded that the use of AT in a tertiary care hospital was 89.5%. The people who had visual, locomotor, hearing, and self-care challenges indicated increased usage of assistive products. Another study that reported the same increase in usage in functional impairment is the rATA conducted in Brazil, whereby an assistive product usage rate of 72.9% was reported. Such data are slightly higher in comparison with the GReAT values range (2.9%-68%) [6, 25]. The AT usage was different among other developing countries, with rates of 7.2% in Pakistan and 11% in Bangladesh. Nevertheless, the usage rate of the AT in the tertiary care facility in North India was significantly higher in comparison with Pakistan and Bangladesh [24,25], but comparable to Brazil's, since the selection criterion of the participants in this study was only the functionally impaired individuals in comparison with the general population. The current research indicated that spectacles, shower/bath/toilet chairs, and lower-limb orthoses were the most commonly used assistive products. The predominance of visual problems is a factor in the popularity of the most widely used product, which is spectacles; the extensive supply of spectacles, both in optical stores and many individual eye care centers; and the adoption of a national presbyopia correction program.

The findings indicate that the government's efforts on the delivery of AT must be reinforced through the contribution of different national and international organizations. The involvement of the industries, as well as the use of public-private partnerships, may also be crucial to enhancing accessibility to AT. In India, the specialized AT services tend to be centered in the larger metropolitan cities and mostly involve mobility and vision services. To seal these loopholes and guarantee more access to AT, non-governmental organizations and community-based services may be required.

## Conclusions

This study demonstrates a significant association between access to and utilization of AT and the levels of both met and unmet AT needs among the participants. Based on the findings of this study, strengthening screening and early identification of AT needs at healthcare facilities is recommended to ensure the timely provision of appropriate assistive products. Improving awareness regarding available government schemes and assistive technology services may help reduce unmet needs. Additionally, strengthening service delivery systems and ensuring the availability and affordability of assistive products at healthcare facilities could improve access and user satisfaction.

The barriers related to affordability and limited awareness identified in this study highlight the need for strengthening the implementation of existing policy initiatives in India. Programs such as the ADIP Scheme aim to improve the affordability of assistive products, while the Rights of Persons with Disabilities Act, 2016, provides a legal framework to promote accessibility and inclusion. Strengthening awareness, outreach, and effective implementation of these mechanisms may help reduce barriers to AT access. The growing recognition of the importance of assistive products as an indicator of rights related to disability and rehabilitation in healthcare systems adds another dimension to the importance of the timely development and implementation of the WHO's rATA tool. Evidence from this study will add value to further guide the design and strengthening of systems geared towards the goal of equitable access to AT and achievement to fulfill the gap between met and unmet need and availability for individuals with disabilities and functional impairments.
